# An EBV recombinant deleted for residues 130-159 in EBNA3C can deregulate p53/Mdm2 and Cyclin D1/CDK6 which results in apoptosis and reduced cell proliferation

**DOI:** 10.18632/oncotarget.7502

**Published:** 2016-02-19

**Authors:** Sanket Kumar Shukla, Hem Chandra Jha, Darine W. El-Naccache, Erle S. Robertson

**Affiliations:** ^1^ Department of Otorhinolaryngology and Tumor Virology Program, Abramson Cancer Center, Perelman School of Medicine at the University of Pennsylvania, Philadelphia, PA-19104, USA

**Keywords:** EBV, EBNA3C, homologous recombination, cell proliferation, apoptosis

## Abstract

Epstein-Barr virus (EBV), a gamma herpes virus is associated with B-cell malignancies. EBNA-3C is critical for *in vitro* primary B-cell transformation. Interestingly, the N terminal domain of EBNA3C which contains residues 130–159, interacts with various cellular proteins, such as p53, Mdm2, CyclinD1/Cdk6 complex, and E2F1. In the current reverse genetics study, we deleted the residues 130-159 aa within EBNA3C open reading frame (ORF) by BACmid recombinant engineering methodology. Our experiments demonstrated that deletion of the 130-159 aa showed a reduction in cell proliferation. Also, this recombinant virus showed with higher infectivity of human peripheral blood mononuclear cells (PBMCs) compared to wild type EBV. PBMCs- infected with recombinant EBV deleted for 130-159 residues have differential expression patterns for the p53/Mdm2, CyclinD1/Cdk6 and pRb/E2F1 pathways compared to wild type EBV-infected PBMCs. PBMCs infected with recombinant virus showed increased apoptotic cell death which further resulted in activation of polymerase 1 (PARP1), an important contributor to apoptotic signaling. Interestingly, cells infected with this recombinant virus showed a dramatic decrease in chromosomal instability, indicated by the presence of increased multinucleation and micronucleation. In addition infection with recombinant virus have increased cells in G0/G1 phase and decreased cells in S-G2M phase when compared to wild type infected cells. Thus, these differences in signaling activities due to 29 amino acid residues of EBNA3C is of particular significance in deregulation of cell proliferation in EBV-infected cells.

## INTRODUCTION

Epstein–Barr virus (EBV), a ubiquitous herpes virus, infects approximately 90-95% of the world's population and is associated with various malignant tumors, including Burkitt's lymphoma, nasopharyngeal carcinoma, and B-cell lymphoma in immune deficient individuals [1]. EBV can infect the cells in either a latent or a lytic manner. Most of the cells are infected latently with EBV and only a small number of the total viral genes are expressed. Viral DNA is maintained in the episomal state and replicates using the host cell DNA polymerase. Infectious virus particles are produced during the lytic reactivation that ultimately causes cell death [2]. EBV has the potential to transform human B-lymphocytes in vitro by maintaining a continuous proliferative state, known as “immortalization” leading to generation of lymphoblastoid cell lines (LCLs) [3]. The LCLs, which are produced in culture carry the viral genome as extra-chromosomal episomes and express nine latent EBV proteins including, the six nuclear antigens (EBNA1, 2, 3A, 3B, 3C & LP), an additional three membrane associated proteins (LMP1, LMP2A & 2B), and the twenty-nine EBV-encoded small RNAs and microRNAs BARTs [4]. These viral factors contribute to activation of the quiescent B-cells from G_0_ into the cell cycle, and to sustain proliferation and maintenance of the viral genome [5].

Among the potential EBV latent antigens, EBNA3A, EBNA3B, and EBNA3C are sequentially encoded in the EBV genome and generate protein products of approximately 1,000 aa. These latent proteins are also essential for EBV to drive primary human B lymphocytes into continuously proliferating LCLs and for maintaining LCL growth [6]. Notably, EBV nuclear antigen 3C (EBNA3C) plays a regulatory role in the transcription of several viral and cellular genes [7].

Cancer development critically depends on the subtle balance between cell proliferation and apoptosis-mediated cell death. The amino- terminal domain of EBNA3C specifically, 130-159 amino acids bind to various proteins which substantially disrupt the coordination of cell proliferation and apoptosis which drives oncogenic transformation. Some of these include p53, Mdm2, Pim-1, CyclinD1, CyclinA and IRF-4 [8, 9, 10, 11, 12]. These molecules can deregulate the cell cycle as well as inhibit apoptosis which can lead to oncogenic transformation of B cells [13].

Previously, we showed that residues 130-190 of EBNA3C strongly stabilizes Mdm2 [8]. Importantly, EBNA3C simultaneously binds to both Mdm2 and p53 and can form a stable ternary complex [9]. Further we also showed that EBNA3C enhances the intrinsic ubiquitin ligase activity of Mdm2 toward p53, which in turn facilitated p53 degradation and control of cell cycle. Besides the p53/Mdm2 complex [12], other complexes such as CyclinD1/Cdk6 important for cell cycle regulation was also stabilized by residues 130-159 of EBNA3C [8]. Together with its binding partners Cdk6, CyclinD1 forms active complexes like CyclinD1/Cdk6 which facilitates cell-cycle progression by phosphorylating and inactivating Rb. This subsequently releases the repression of E2F1 target genes and permits the cell cycle progression.

In the field of herpesvirus, homologous recombination are widely applied tools for generating mutants [14]. The use of bacterial artificial chromosome (BAC) technology in herpesvirus genetics has made their genomes accessible to the tools of bacterial genetics [15]. Previously, we successfully constructed an EBVBAC system with a GFP tag [16]. The recombinant virus not only successfully infected human peripheral B-cells, but also expressed GFP signal during early primary infection making it a useful reagent for infection studies [16]. Interestingly, recombinant virus was found to activate the CD40 receptor in a time dependent manner [16].

In this report, we generated a recombinant virus by using *galK* positive/negative selection to delete residues 130-159 within the N terminal domain within EBNA3C open reading frame (ORF). This recombinant virus were examined to delineate the role of EBNA3C, and its binding domain for p53/Mdm2, CyclinD1/Cdk6 and pRb/E2F1 in B-cell proliferation and activation during latent and primary infection.

## RESULTS

### Generation of a recombinant BACEBV-GFP deleted for residues 130-159 of EBNA3C

**Figure 1:**
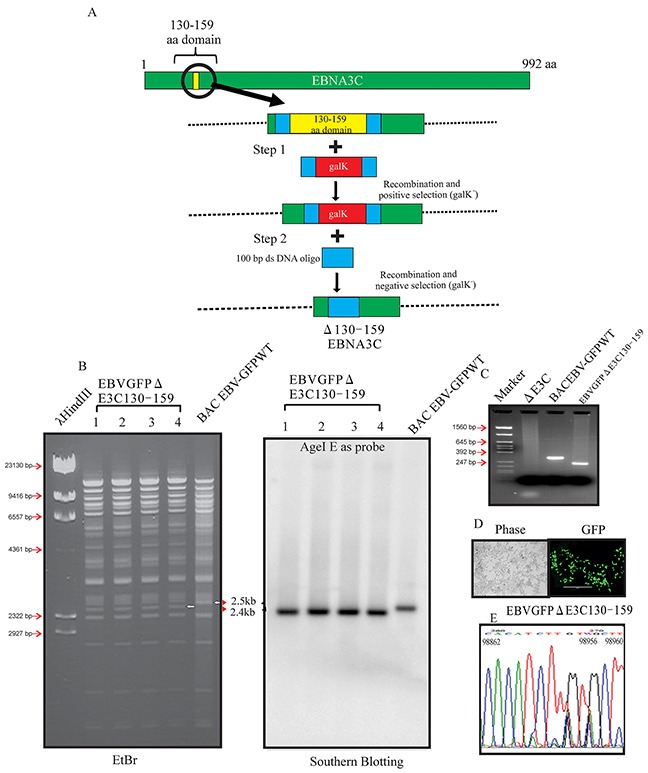
Generation of the recombinant viruses EBVGFPΔE3C130-159 **A.** Schematic diagram showing generation of EBVGFPΔE3C130-159, a recombinant BACEBV-GFP with deletion of the residues 130-159 of EBNA3C. **B.** Ethidium bromide-stained gel and Southern blots with BACEBV-GFPWT (lane 6) and the mutated BACmid, EBVGFPΔE3C130-159, cleaved with AgeI (Lanes 2–5). **C.** PCR analysis for EBVGFPΔE3C130–159 recombinant virus at the junction of the deletion within EBNA3C. **D.** Cells were transfected with EBVGFPΔE3C130–159. GFP expression levels were monitored by fluorescent microscopy 2 days after transfection. **E.** Selected chromatogram of the junction PCR products. Number indicates the position of sequence at B95.8 EBV genome.

Our previous studies showed that EBNA3C contributes to proliferation of EBV-associated lymphomas [11, 17, 18, 19]. The p53/Mdm2 and Cyclin D1/Cdk6 binding site within EBNA3C are located in the amino-terminal residues 130-190 aa of EBNA3C. This binding site were shown to be associated with EBV growth and proliferation [8, 12]. However, no further investigation were performed within the viral genome. Here we constructed Δ130-159 EBNA3C recombinant virus, on the backbone of the BACEBV-GFP, a GFP tagged EBV generated previously [16]. The BACEBV-GFPWT carries the EBV genome, a GFP tag and resistance genes for ampicillin, kanamycin and puromycin [16]. Infectious EBV can be produced by transfection of BACEBV-GFPWT into HEK-293T cells, selection followed by chemical induction [16]. We used a homologous recombination system in sw102, a modified *E.coli* strain and a *gal*-positive/negative selection method [20], which has been described in additional methods, to introduce the desired mutation into BACEBV-GFPWT. Briefly, we have adopted the *gal* selection method to first insert the *gal* expression cassette into the coding region of BACEBV-GFPWT (Figure 1A). In the second step, the *gal* cassette is substituted by the DNA fragment containing the 50 bp upstream and 50 bp downstream of the EBNA3C 130-159 region ORF (100bp). Thereafter, *gal*-negative clones were selected by resistance to 2-deoxy-galactose (DOG) on minimal plates with glycerol as the carbon source. The resulting BAC recombinants were screened and analyzed on 0.65% agarose and Southern blot analysis to show that the specific domain was removed from the EBV genome (Figure 1B and 1C). Digestion of the BACEBV-GFPWT with AgeI generated a 2.4kb fragment compared to 2.5kb of the full-length EBNA3C domain suggesting that the 130-159 domain was removed at the desired site (Figure 1B and 1C). To further re-confirm whether the altered digestion pattern of the BAC mutant was the result of expected recombination, we performed PCR across the junction by using the primers designed at the recombination site. The result showed that the PCR bands from EBNA3C ORF shifted based on the presence of deletion (Figure 1C). Finally, the PCR products were sequenced to confirm the expected deletion (Figure 1D and 1E).

### Generation of the EBNA3C recombinant virus

**Figure 2:**
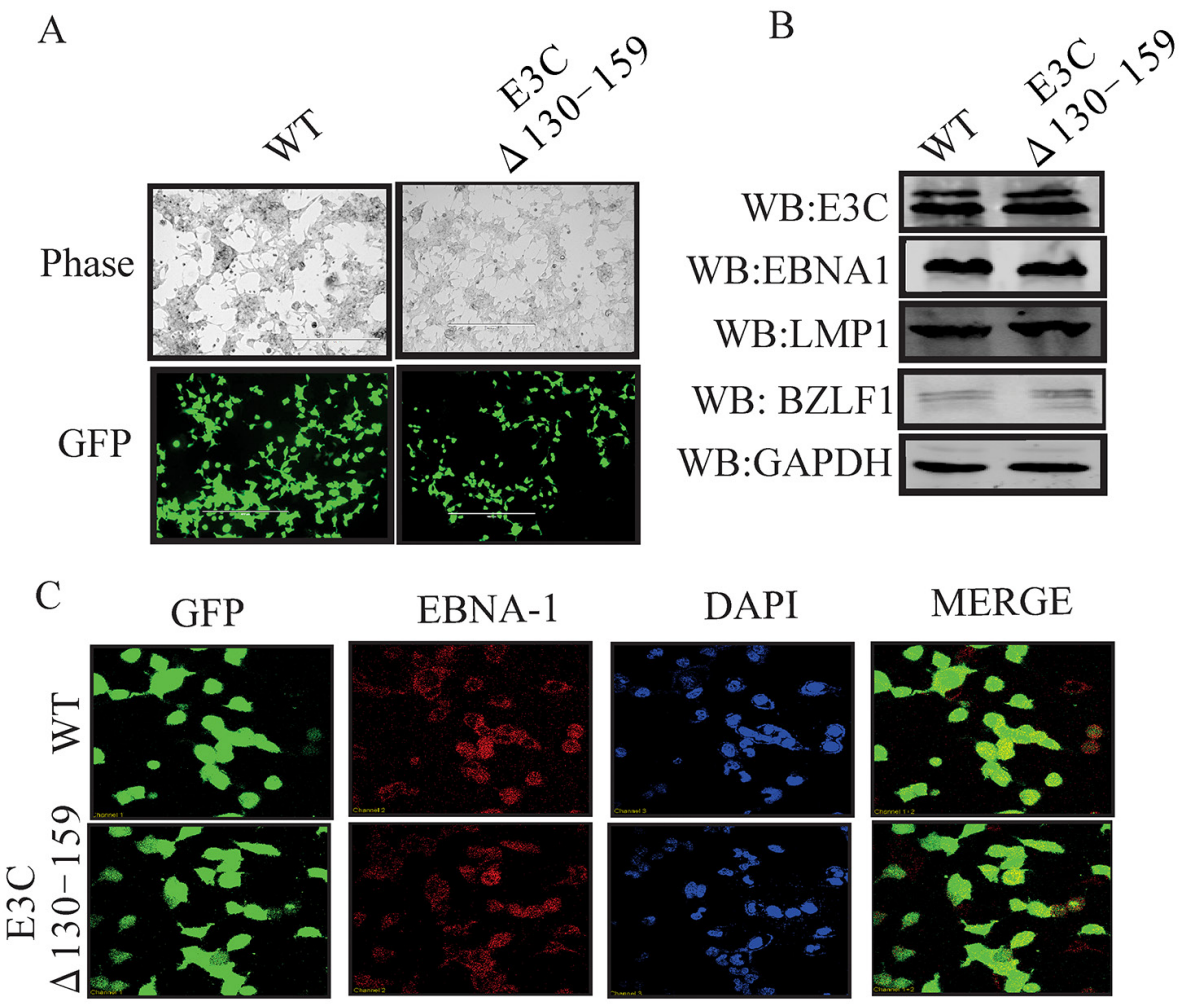
Characterization of EBVGFPΔE3C130-159 stable 293T cell lines **A.** HEK-293T cells were transfected with EBVGFPΔE3C130-159 DNA. The transfected cells were split and selected with puromycin. The puromycin-resistant cells were pooled and passed for 3–4 weeks. The homogenous population of GFP-positive cells harboring EBVGFPΔE3C130–159 (ΔE3C130-159) genome were obtained. GFP expression levels were monitored by fluorescent microscopy and compared with BACEBV-GFPWT (WT) stabilized HEK-293T cells **B.** Western blots for EBNA3C, EBNA1, LMP1 and BZLF1 for the BACEBV-GFPWT and EBVGFPΔE3C130-159 stable HEK-293T cell lines. GAPDH was an endogenous control. **C.** Immunofluorescence analysis for EBNA1 in BACEBV-GFPWT and EBVGFPΔE3C130-159 stable HEK-293T cell lines.

To reconstitute the recombinant viruses, we transfected EBVGFPΔE3C130-159 DNA into HEK-293T cells. The transfection efficiencies were monitored by fluorescence microscopy for GFP-positive cells which were detected after 24 to 48 hrs of post-transfection (Figure 1D). The transfected cells were selected by 1 μg/ml puromycin to generate HEK-293T cell lines harboring EBVGFPΔE3C130-159 DNA (Figure 2A). Subsequently, the cells were fixed and immunostained against EBNA1, which confirmed that the EBVGFPΔE3C130-159 DNA stable cell line harbored the EBV genome (Figure 2C). Furthermore, we examined the expression of EBNA3C, EBNA1, LMP1 and BZLF1 expression by Western blot (WB) analysis. The expression patterns of these proteins are shown in Figure 2B. Similar levels of expression for EBNA1, LMP1 and EBNA3C proteins were found in EBVGFPΔE3C130-159 when compared to BACEBV-GFPWT indicating that the mutation had little effect on the stability of EBNA3C. The BZLF1 protein was minimally expressed, suggesting that the stable cell lines are tightly latent with minimal lytic activities (Figure 2B).

### EBNA3C recombinant deleted for residues 130-159 showed reduced cell growth activity

**Figure 3:**
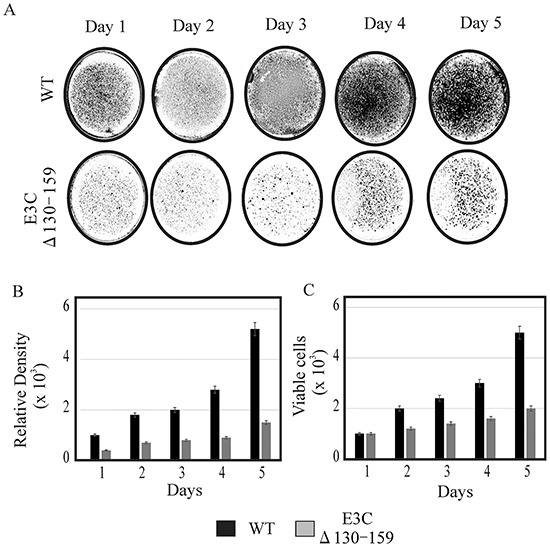
Cell growth assay for BACEBV-GFPWT and EBVGFPΔE3C130-159 stable HEK-293T cell lines **A.** 1×10^3^ BACEBV-GFPWT (WT) and EBVGFPΔE3C130-159 (ΔE3C130-159) stable HEK-293T cell lines were plated in DMEM with 5% FBS and cultured in 37°C incubation with 5% CO_2_. The medium were removed and the plates were washed by 1x PBS. The cells were fixed on the plates with 4% formaldehyde and stained with 0.1% crystal violet. The photographs were acquired by Li-Cor Odyssey. **B.** The relative density was quantitated using Odyssey V 3.0. The number represents the averages of data from three independent experiments. 2-tailed Student's *t*-test was performed to evaluate the significance of differences in the mean values, and *p* values < 0.05 were considered statistically significant and is denoted by an asterisk *. **C.** 1 × 10^3^ million BACEBV-GFPWT and EBVGFPΔE3C130-159 expressing HEK-293T cells were subjected to cell proliferation assays by Trypan blue dye exclusion method.

EBV is a ubiquitous human oncovirus and can induce cellular transformation of infected cells [21, 22]. The cells harboring the viral genome have an enhanced capability for driving cell growth. Therefore, to determine the growth rate of EBVGFPΔE3C130-159, transfected HEK-293T cells were evaluated using cell growth assays with 0.2 million EBVGFPΔE3C130-159 and compared with BACEBV-GFPWT cells. HEK-293T cells were seeded in 100 mm petri dish with DMEM plus 5% BGS and 1 μg/ml puromycin. The dishes were collected at 1, 2, 3 and 4 days, washed and fixed on plates with 4% Para-formaldehyde and stained with 0.1% crystal violet. The cells were scanned using a LiCor Odyssey scanner and the area of cells (pixels) in each dish was calculated using the Odyssey V3.0 software. Our results demonstrated that the number of HEK-293T positive EBVGFPΔE3C130-159 cells were reduced by 40-70% compared to BACEBV-GFPWT HEK-293T positive cells at day 3 and 4 (Figure 3A). Therefore, residues 130-159 which binds p53/Mdm2 and CyclinD1/Cdk6 complex is important for EBV-mediated cell growth. We further supported our observations by performing cell proliferation assays. Our data demonstrated that cell proliferation was significantly reduced by greater than 50% by 5 days with Δ130-159 recombinant when compared with BACEBV-GFPWT infected cells (Figure 3C). Therefore, these residues are important for promotion of cell growth and proliferation.

### The recombinant virus EBVGFPΔE3C130-159 can infect human PBMCs

**Figure 4:**
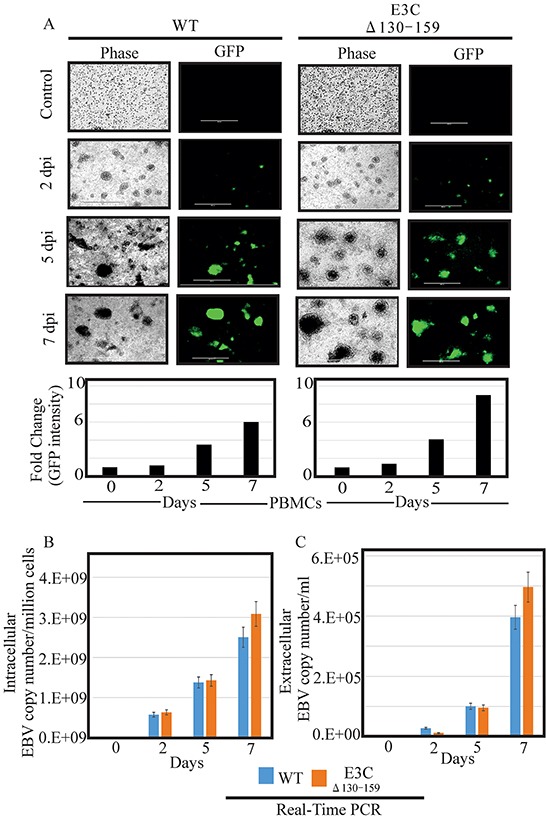
Comparisons of relative infectivity for BACEBV-GFPWT and EBVGFPΔE3C130-159 recombinant virus **A.** PBMCs were infected by BACEBV-GFPWT (WT), and EBVGFPΔE3C130-159 (ΔE3C130-159) virus with equally loading. GFP expression was monitored under a fluorescent microscope after 2 dpi, 5 dpi and 7 dpi. **B.** Intracellular EBV virion progenies copies were measured by a real-time PCR with primers to EBNA1 at 2 dpi, 5 dpi and 7 dpi. **C.** Extracellular EBV virion progenies were analyzed by a real-time PCR with primers to EBNA1 at 2 dpi, 5 dpi, and 7 dpi. Dpi, days post infection.

Earlier, studies showed that BACEBV-GFPWT was highly competent for infecting PBMCs [16]. Here we determined whether this recombinant virus possess the ability to efficiently infect human PBMCs *in vitro*. BACEBV-GFPWT and EBVGFPΔE3C130-159 expressing HEK-293T cells were induced in the presence of butyric acid at a final concentration of 3mM and TPA at a concentration of 20 ng/ml [16]. The supernatant from cell culture were collected and treated with DNAse. The viruses were concentrated by ultracentrifugation 70,000xg at 4°C and the virus stocks were quantified by qRT-PCR. Equal virus particles were added to 1×10^6^ PBMCs washed and replaced with complete media. The infected PBMCs were monitored for GFP signal using fluorescence microscopy. The concentrated viruses were used to infect PMBCs and the infected cells were monitored at 2, 5 and 7 days post infection (dpi). Importantly, we have found that virion particles from both BACEBV-GFPWT and recombinant virus was detected as GFP as early as 2 dpi (Figure 4A). Our results showed that EBVGFPΔE3C130-159 infected PMBCs showed detectable higher GFP signals compared to BACEBV-GFPWT at 5 and 7 dpi (Figure 4A). Infected PBMCs were collected at 2, 5 and 7 dpi for further analysis. Viral DNA levels were determined by qRT-PCR. GFP signals reflected increases in both extracellular and intracellular viral copies in a time dependent manner. Surprisingly, the EBV copies was higher consistently for EBV GFPΔE3C130-159 compared to BACEBV-GFPWT at 7dpi (Figure 4B and 4C). The differences were higher but not significant. This suggests that the EBVGFPΔE3C130-159 have higher infectivity in PBMCs. We have also confirmed our findings with collected supernatant from primary infection to infect fresh new PBMCs using similar number of viral progeny (Supplementary Figure S1).

### Analysis of EBV latent/lytic proteins expression in PBMCs infected with EBVGFP ΔE3C130-159

**Figure 5:**
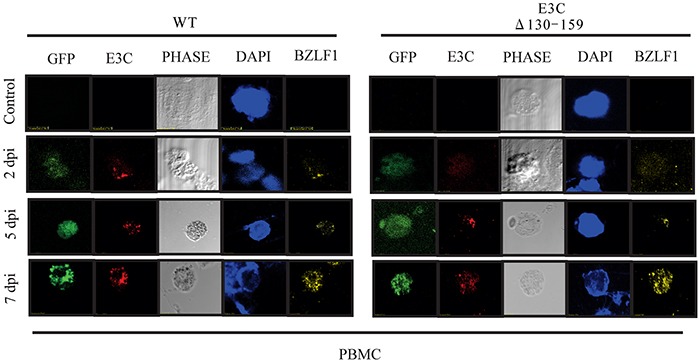
EBV latent and lytic gene expression during early stage of infection Immunofluorescence assay for PBMCs infected by BACEBV-GFPWT (WT) and EBVGFPΔE3C130-159 (ΔE3C130-159) virus at 2 dpi, 5 dpi and 7 dpi. Infected PBMCs at 2, 5 and 7 dpi were stained for EBNA3C and BZLF1 protein expression. PBMCs expressed GFP, indicating the presence of viral genome.

To evaluate the gene expressions pattern during early events of infection, EBVGFP ΔE3C130-159 was used to infect PBMCs and latent/lytic gene expression were analyzed (Figure 5). Further, the expression were compared with BACEBV-GFPWT infection. Latent expression of EBNA3C, and the immediate early transactivator (BZLF1) gene were monitored by immunofluorescence at 2, 5 and 7 dpi (Figure 5). Expression of EBNA3C and BZLF1 were weakly detectable at 48 hrs, and strongly detected at 5 and 7 dpi. Both the proteins were consistently detected through 7 dpi. However, we found similar levels of expression up to 7 dpi, when comparing BACEBV-GFPWT and the recombinant EBVGFPΔE3C130-159 deleted EBV infections.

### EBV recombinant for EBNA3C deregulates the expression of p53/Mdm2 and CyclinD1/Cdk6 complexes

**Figure 6:**
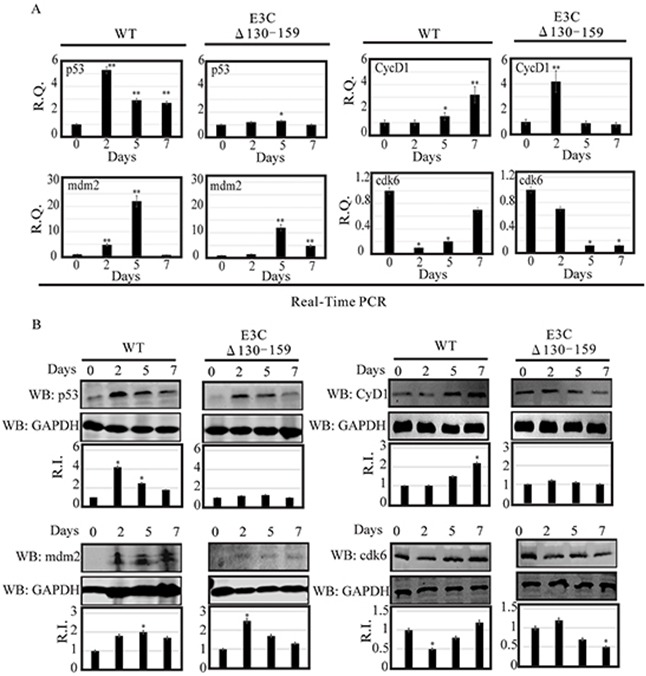
Analysis of mRNA and protein levels for p53, Mdm2, CyclinD1 and Cdk6 during EBV primary infection at 2, 5, and 7 dpi **A.** Human PBMCs were infected by BACEBV-GFPWT (WT) and EBVGFPΔE3C130-159 (ΔE3C130-159) virus and cells were harvested at 2, 5, and 7 days p.i. Total RNAs were extracted by using TRIzol (Invitrogen), and cDNAs were synthesized using a high capacity RNA-to-cDNA kit. The mRNA levels of p53, Mdm2, CyclinD1 and Cdk6 were quantified by qRT-PCR on a StepOnePlus real-time PCR system. **B.** The protein levels of p53, Mdm2, CyclinD1 and Cdk6 in PBMC infected with BACEBV-GFPWT and EBVGFPΔE3C130-159 virus at 2, 5 and 7 days p.i. were analyzed Western blot. dpi, days post-infection; RQ, relative quantity; RI, relative intensity. *P<0.05; **P<0.001 by Student's t test compared to control.

Earlier, using both *in vitro* and *in vivo* binding experiments we had showed that EBNA3C physically interacted with p53 through residues 130–190 [23]. This interaction blocked p53 dependent transcriptional activation and subsequent apoptotic induction [24]. This region also physically interacted with Mdm2 via its central acidic domain [12]. This interaction is important for recruitment of Mdm-E3 ligase activity which led to degradation of p53 [12]. Here, we examined the expression levels of p53 and Mdm2 in BACEBV-GFPWT and EBVGFPΔE3C130-159 virus infected primary cells [12]. Our result showed that in BACEBV-GFPWT infection, the p53 transcript expression was increased from 2 dpi (5.2 fold) and gradually decreased at 7 dpi (2.3 fold), compared to control (Figure 6A). However, in PBMCs infected with EBVGFPΔE3C130-159 virus, the p53 transcript showed a small increase from 2 dpi to 5 dpi and was reduced at 7 dpi (Figure 6A). The WB analysis also supported the result of qRT-PCR where p53 expression was gradually decreased from 2 dpi (4.2 fold) to 7 dpi (1.8 fold) in BACEBV-GFPWT infection (Figure 6B). In EBVGFPΔE3C130-159 infection, almost similar levels of expression was found at 2 and 5 dpi which was ultimately down-regulated at 7 dpi (0.8 fold) (Figure 6B).

As discussed previously, the targeted degradation of p53 by one of its negative regulators, Mdm2, represents a critical circuit in the regulation of p53-mediated tumor suppressive functions [12]. Therefore, we also checked the expression of Mdm2 in PBMCs infected with BACEBV-GFPWT and EBVGFPΔE3C130-159 virus. Our result showed that in BACEBV-GFPWT infection, mdm2 transcripts were found to be upregulated from 2 dpi (4.9 fold) to 5 dpi (22 fold) and get down regulated at 7 dpi (0.9 fold) (Figure 6A). Although the expression of mdm2 was less in EBVGFPΔE3C130-159 virus at 2 dpi (1.2 fold) and 5 dpi (12 fold), the trend was similar as we found in BACEBV-GFPWT infection (Figure 6A). We also confirmed the qRT-PCR results with the WB analysis. A similar trend of Mdm2 protein expression was found in BACEBV-GFPWT infection (Figure 6B), as we found at transcript level. However, in contrast, Mdm2 protein expression was upregulated at 2 dpi (2.5 fold) and was gradually down-regulated at 5 dpi (1.70 fold) to 7 dpi (1.45) in EBVGFPΔE3C130-159 virus infected cells (Figure 6B).

Previously, we demonstrated that EBNA3C forms a complex with CyclinD1 in human cells [8]. Detailed mapping experiments showed that aa 130–159 of EBNA3C binds to two different sites of CyclinD1. We further showed that EBNA3C together with CyclinD1/Cdk6 complex also efficiently nullifies the inhibitory effect of pRb on cell growth [8]. Since the 130-159 E3C domain is important for stabilization of CyclinD1/Cdk6 complex. Hence, it would be interesting to monitor the expression level of CyclinD1/Cdk6 in PBMCs infected with EBVGFPΔE3C130-159. Our result showed that the cyclinD1 transcript levels were upregulated at 2 dpi (4.2 fold) and then down-regulated at 5 dpi (0.9 fold) to 7 dpi (0.7 fold) in PBMCs infected with EBVGFPΔE3C130-159 (Figure 6A). However, in BACEBV-GFPWT infection CyclinD1 level was consistently upregulated from 2 dpi (1 fold) to 7 dpi (3.2 fold) (Figure 6A). WB also supported these findings where CyclinD1 protein levels were consistently down-regulated from 2 dpi (1.26 fold) to 7 dpi (1 fold) in EBVGFPΔE3C130-159 infected cells and upregulated in BACEBV-GFPWT infected cells (Figure 6B). A similar trend was seen for Cdk6 expression (Figure 6A and 6B).

### EBV recombinant 130-159 for EBNA3C differentially regulates the pRb/E2F1 mediated signaling pathway

**Figure 7:**
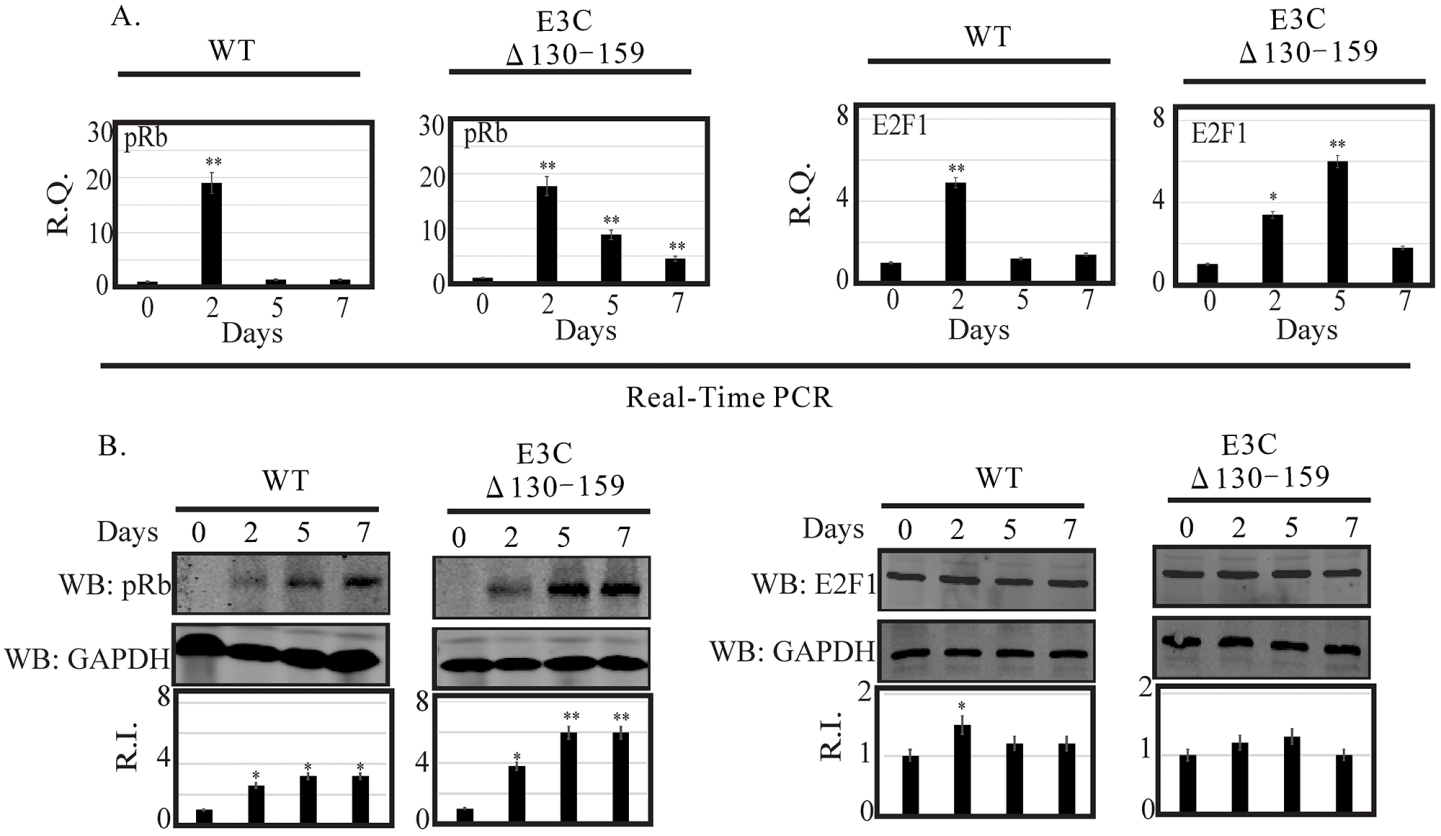
Analysis of mRNA and protein levels for pRb and E2F1 during EBV primary infection at 2, 5, and 7 dpi **A.** Human PBMCs were infected by BACEBV-GFPWT (WT) and EBVGFPΔE3C130-159 (ΔE3C130-159) virus and cells were harvested at 2, 5, and 7 days p.i. Total RNAs were extracted by using TRIzol (Invitrogen), and cDNAs were synthesized using a high capacity RNA-to-cDNA kit. The mRNA levels of pRb and E2F1 were quantified by qRT-PCR on a StepOnePlus real-time PCR system. **B.** The protein levels of pRb and E2F1 in PBMC infected with BACEBV-GFPWT and EBVGFPΔE3C130-159 virus at 2, 5 and 7 days p.i. were analyzed Western blot. dpi, days post-infection; RQ, relative quantity; RI, relative intensity. *P<0.05; **P<0.001 by Student's t test compared to control.

DNA damage signaling events are clearly involved in the induction of E2F1 and its stabilization through several post-translational modifications [25]. However, the mechanism by which these modifications can lead to E2F1 stabilization remains unclear. E2F1 is regulated through a ubiquitin-proteasome pathway in a cell cycle-dependent manner, which relies upon its dissociation from its regulatory partner, pRb tumor suppressor [17]. Previous studies suggested that the N-terminal domain of EBNA3C may be critically involved in disrupting the CyclinD1/Cdk6-pRb-E2F1 as well as p53-E2F1-pRb signaling pathway to enhance cell cycle or apoptotic activities [8, 17]. Moreover, it has also been showed that Mdm2, which was previously shown to be regulated by residues 130-159 of EBNA3C, can also interact with pRb/E2F1 and modulates its activities [8]. In this study we investigated the expression level of pRb and E2F1 in BACEBV-GFPWT and EBVGFPΔE3C130-159 virus infected primary cells. Our result showed that the pRb transcript was upregulated at 2 dpi (25 fold) and down regulated from 5 dpi (8 fold) to 7 dpi (4 fold) in EBVGFPΔE3C130-159 infected cells (Figure 7A). A similar patterns were observed for BACEBV-GFPWT infected cells (Figure 7A). Surprisingly, there was significant reduction of pRb expression at 5 (1.36 fold) and 7 dpi (1.30 fold) when compared to EBVGFPΔE3C130-159 virus infection at 5 and 7 dpi (8 and 4 fold respectively). WB also found similar trends for pRb protein expression in both BACEBV-GFPWT and EBVGFPΔE3C130-159 virus infected cells (Figure 7B).

The apoptotic phenomena due to downregulation of EBNA3C expression in LCLs is attributed as a cumulative effect of both E2F1 and p53 mediated apoptosis [17]. The inhibitory effect of the N-terminal domain of EBNA3C on E2F1 mediated transcriptional activity led us to further investigate the basal expression levels of E2F1 in primary infection model systems. In order to determine a definitive role for residues 130-159 of EBNA3C in attenuating this E2F1 mediated DNA damage response, we infected PBMCs with EBVGFPΔE3C130-159 virus to analyze E2F1 transcripts during early infection (Figure 7A). Interestingly, EBVGFPΔE3C130-159 virus infected cells displayed a drastic increase in E2F1 activation from 2 (3.4 fold) to 5 dpi (6.0 fold) when compared to uninfected PBMCs (Figure 7A). A similar trend, however to a lesser extent (1.4 fold) was observed at 7 dpi. However, in BACEBV-GFPWT infection, the E2F1 transcript levels was seen particularly robust at 2 dpi while gradually declined to an expression level similar to uninfected PBMCs (Figure 7A). These data clearly supported our hypothesis that EBNA3C residues 130-159 are important for regulation of genotoxic stress during early stages of infection. To support the gene expression profiles obtained with qRT-PCR, we also determined the protein levels of E2F1 in PBMCs infections. Infection with EBVGFPΔ130-159 virus also increased E2F1 protein, especially at 2 and 5 dpi (1.2 and 1.25 fold change respectively), but the levels were down regulated at 7 dpi (1.00 fold) consistent with the mRNA levels determined by qRT-PCR (Figure 7B).

### Enhanced apoptotic cell death and PARP-1 cleavage was found in B cell infected with EBVGFPΔE3C130-159

**Figure 8:**
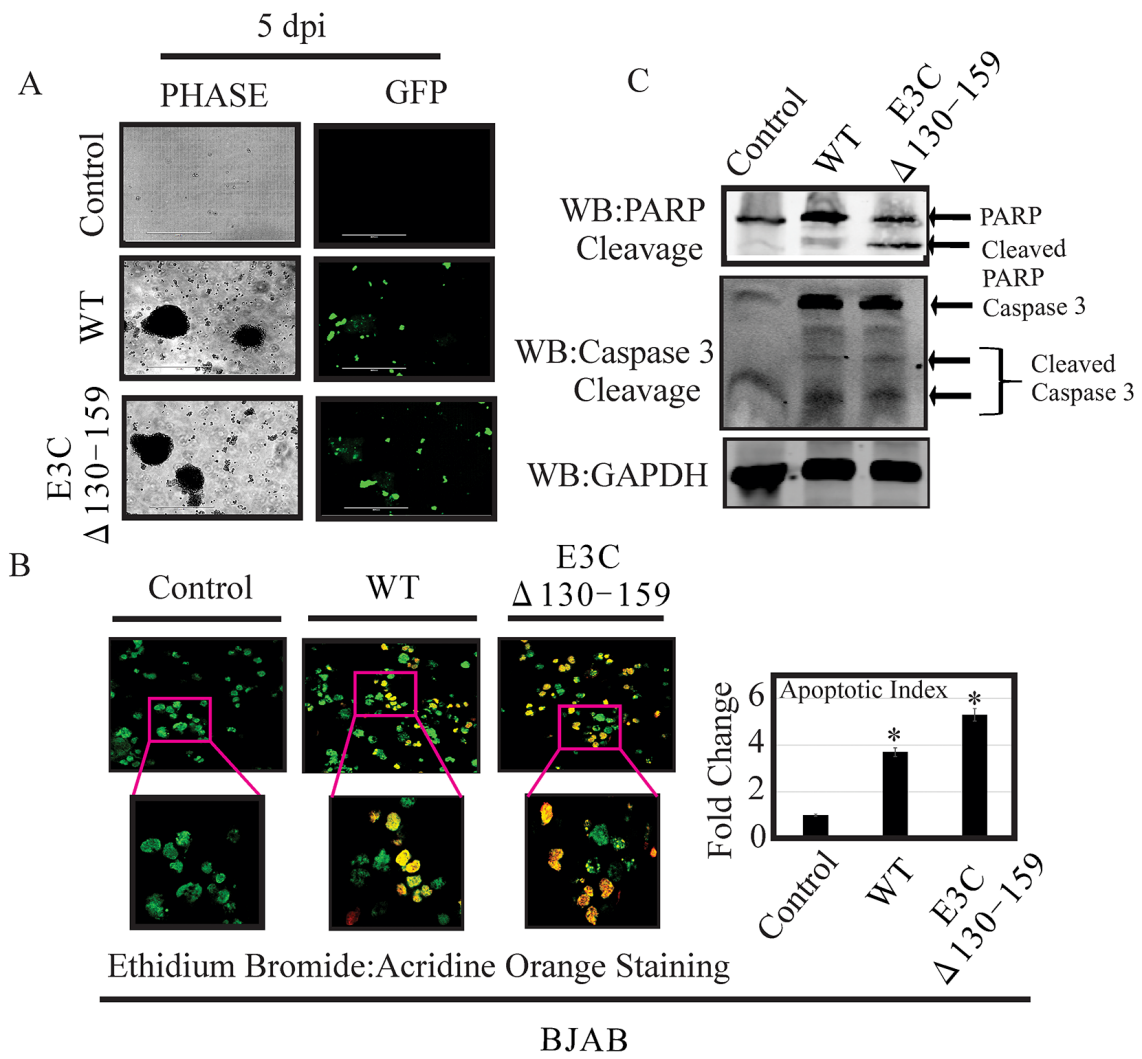
EBVGFPΔE3C130-159 virus infected cells can enhance induction of apoptosis **A.** BJAB Cells infected with BACEBV-GFPWT (WT) and EBVGFPΔE3C130-159 (ΔE3C130-159) virus. GFP signal was monitored for the confirmation of infection. **B.** After 5 days p.i. cells were stained with a mixture of ethidium bromide and acridine orange. Fluorescence microscopy was used to analyze the cells. Pictures were captured through three channels, green, red, and yellow, which represent live cells, dead cells, and apoptotic cells respectively. For each slide, 10 fields (each containing 40 to 100 cells) were captured and counted. Experiments were performed 3 times. **C.** BJAB cells uninfected and infected with EBVGFPΔE3C130-159 virus cells compared to a BACEBV-GFPWT infection were evaluated for PARP1 and Caspase 3 cleavage along with GAPDH as control. *P<0.05; by Student's t test compared to control.

Apoptosis, or programmed cell death, is a complex mechanism by which a cell regulates its own destruction to control the process of cell proliferation [26]. The N-terminal 130-159 aa of EBNA3C can regulate p53. Therefore we checked apoptotic cell death in BJAB cells infected with EBVGFPΔE3C130-159 virus and compared with BACEBV-GFPWT virus infection (Figure 8A). To specifically visualize the apoptotic cells and quantitatively distinguished them from necrotic cells, we performed apoptosis assays using ethidium bromide and acridine orange [27, 28]. Cells are classified into three different groups, i.e., apoptotic, necrotic, and live cells [28]. The results clearly demonstrated that the cells infected with EBVGFPΔE3C130-159 virus were more prone to apoptotic induction than cells with BACEBV-GFPWT virus (Figure 8B). There was 1.6 fold rise in apoptotic levels when compared to BACEBV-GFPWT virus (Figure 8B). Together, these results further strengthened our findings that EBVGFPΔE3C130-159 virus can lead to diminish cell proliferation, which is perhaps mediated in part through enhancement of cellular apoptosis. Further, we investigated the effect of EBVGFPΔE3C130-159 infection on the expression of proteins involved in the apoptotic pathway. First, the expression of PARP1, which functions in DNA repair, was analyzed. PARP1 cleavage was higher in EBVGFPΔE3C130-159 virus infected cells compared to BACEBV-GFPWT virus infected BJAB (Figure 8C). The Caspase-3 expression was barely detectable in uninfected BJAB cells, but was induced following infection by both BACEBV-GFPWT and EBVGFPΔE3C130-159 viruses. The expression levels of Caspase3 cleavage did not show significant differences between BACEBV-GFPWT and EBVGFPΔE3C130-159 virus infected BJAB cells (Figure 8C). This may suggest utilization of a caspase independent apoptotic pathway for EBNA3C residues 130-159.

### EBNA3C residues 130-159 differentially modulates chromosomal instability in BJAB cells

**Figure 9:**
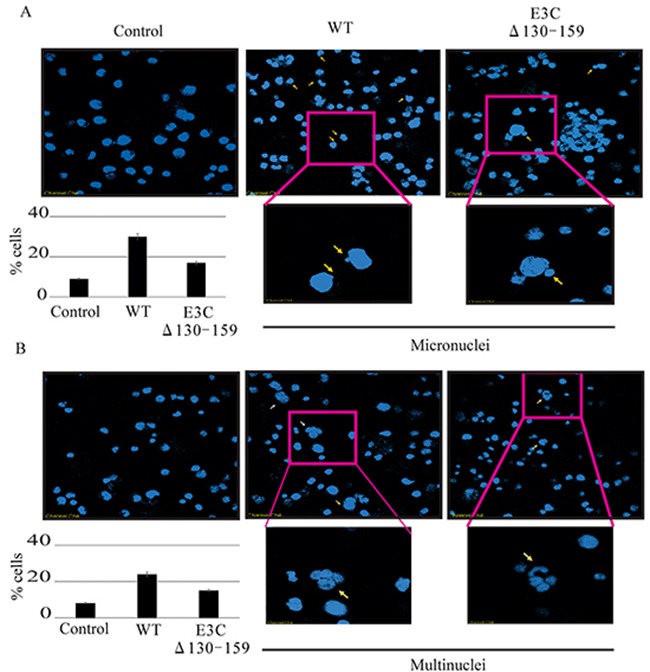
Induction of micronuclei and multinucleation in BJAB cells infected with BACEBV-GFPWT and EBVGFPΔE3C130-159 virus **A.** DAPI staining of representative fields shows increased micronuclei (yellow arrows). **B.** multinucleation (white arrows) in BJAB cells infected with BACEBV-GFPWT (WT) and EBVGFPΔE3C130-159 (ΔE3C130-159) virus.

Chromosomal instability, a particular hallmark of human cancer, has been considered an adaptive response of cancer cells to environmental pressure [29, 30]. In most cases, chromosomal alterations, as well as additional structural alterations such as micro and multinucleation formation [30]. A number of studies have suggested that the loss of p53 function plays a critical role in the preservation of chromosomal instability [31, 32]. Since residues 130-159 of EBNA3C is important for regulating p53 function we compared the chromosomal instability between BACEBV-GFPWT and EBVGFPΔE3C130-159 virus infected BJAB cells. In uninfected BJAB cells about 9% of cells showed micronuclei (Figure 9A). In contrast, the cells with BACEBV-GFPWT infection displayed increased micronuclei (30%). Interestingly cells infected with EBVGFPΔE3C130-159 virus had 17% (Figure 9A) of this phenotype which was significantly less when compared to BACEBV-GFPWT infection. On the other hand the multinucleated cells were found in 8% of un infected BJAB cells, whereas, 24% in BACEBV-GFPWT and 17% in EBVGFPΔE3C130-159 virus infection (Figure 9B). These results indicate that 130-159 aa domain of EBNA3C is crucial for p53 regulation as well as chromosomal instability.

### EBNA3C residues 130-159 are important for G1 to S transition in EBV-infected PBMCs

**Figure 10:**
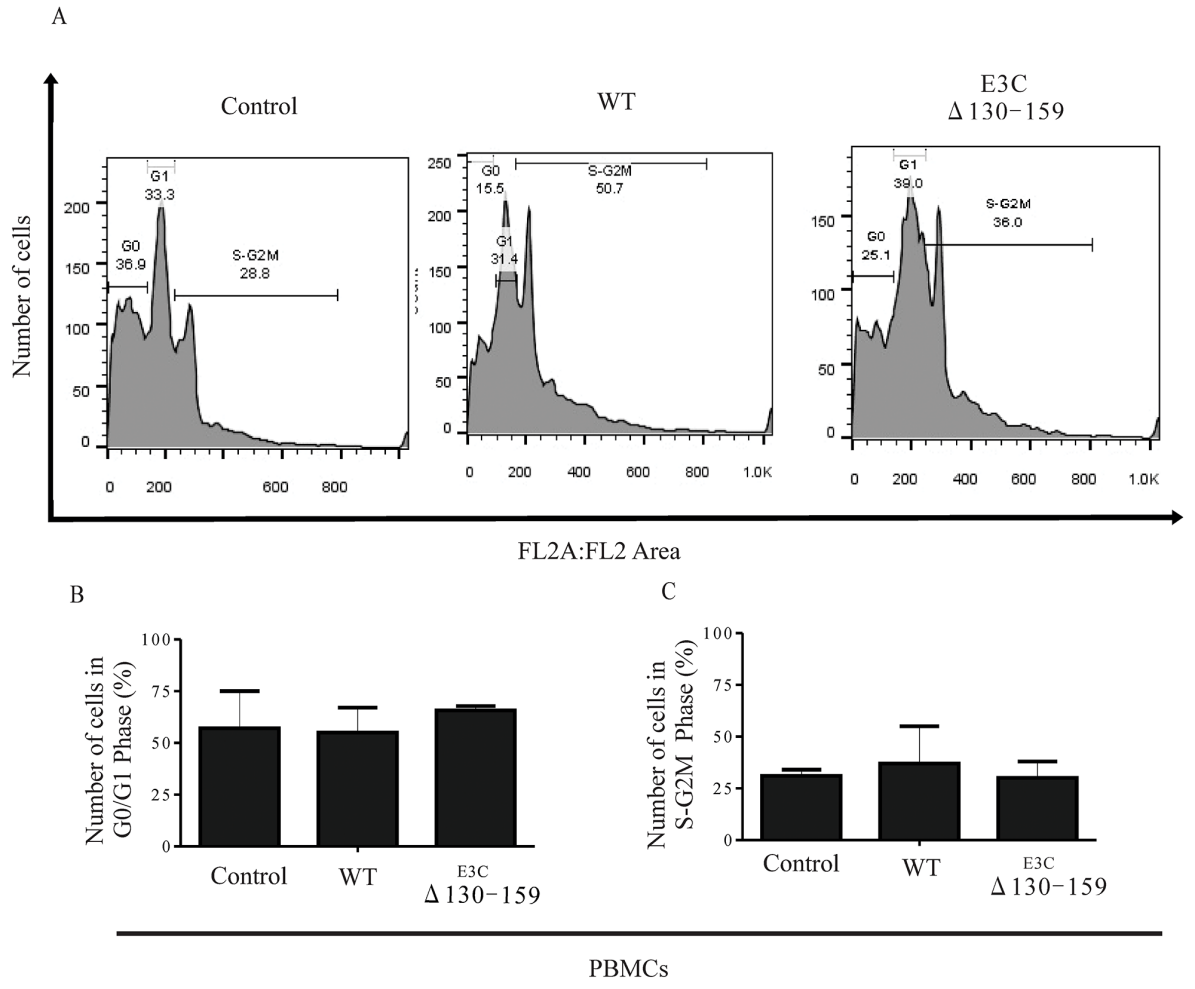
EBNA3C residues 130-159 of EBV is critical for G1 to S phase progression **A.** Human PBMCs were grown for 12 h in RPMI medium containing 10% FBS (+serum) or 0.1% FBS (−serum). Further the cells were infected with BACEBV-GFPWT (WT) and EBVGFPΔE3C130-159 (ΔE3C130-159) virus. Propidium iodide stained cells were analyzed by flow cytometry. **B.** and **C.** The bar diagram represents the change in cell cycle profile in G1 and S-G2M.

Earlier, It was shown that residues 130-159 of EBNA3C can interact with CyclinD1/Cdk6 complex and is important to bypass the G1/S checkpoint in LCLs [8]. To further confirm this finding we infected PBMCs with BACEBV-GFPWT and EBVGFPΔE3C130-159 virus to analyze the cell cycle progression by flow cytometry (Figure 10). Analyses of infected PBMCs with EBVGFPΔE3C130-159 virus revealed an increased percentage of cells at the G0/G1 phase of the cell cycle compared to BACEBV-GFPWT (Figure 10B). Furthermore, a decrease percentage of cells in the S- G2/M phase was seen for the Δ130-159 infected PBMCs compared to BACEBV-GFPWT infected cells (Figure 10C).

## DISCUSSION

EBNA3C interacts with an array of transcription factors, cell cycle regulatory proteins, chromatin remodeling enzymes and ubiquitin-proteasome machinery that eventually leads to B cell lymphomagenesis [9, 12, 17, 24, 34, 35, 36]. Earlier, reports demonstrated that deletion of EBNA3C has a significant effect on LCLs growth and proliferation [35]. We also showed that EBNA3C deleted recombinant viruses had lower infectivity of human primary B-cells which were also reduced for B-cell growth and proliferation [37].

The N-terminal 130-159 of EBNA3C is critical because its binds to important cell cycle regulatory proteins such as p53 and CyclinD1 [8, 24]. After binding with p53, EBNA3C blocks p53 dependent transcriptional activation and subsequent apoptotic induction [24]. This domain also recruits Mdm2-E3 ligase activity towards p53 facilitating its degradation [12]. Apart from p53 and Mdm2 regulation, this domain is also important for regulating CyclinD1/Cdk6 complex [8]. A small N-terminal region which lies between amino acids 130–159 of EBNA3C binds to CyclinD1 [8]. This binding increases kinase activities of CyclinD1/Cdk6 complex, thereby increasing the phosphorylation status of the pRb tumor suppressor protein, which, in turn, facilitates its ubiquitin-proteasome dependent degradation [8].

Additional results showed the importance of the 130-159 region of EBNA3C by using transient transfection assay reporter systems. Here, we investigated the role of these EBNA3C residues by using a reverse genetics approach. We successfully generated a recombinant virus which was more efficient in producing progeny virus. However, similar levels of BZLF1 expression was found both in BACEBV-GFPWT and mutant virus after infection.

In a previous study using a recombinant EBV expressing, conditionally active EBNA3C, Maruo *et al.* further demonstrated the importance of this particular N-terminal region in maintaining LCL outgrowth [23]. This study further showed that deletion of this particular domain was not able to maintain cell proliferation of EBV-transformed LCLs [23]. Therefore, this short stretch of EBNA3C domain is of particular significance in deregulating cell proliferation of EBV-infected cells. We have also found significantly less proliferation for EBVGFPΔE3C130-159 established cell lines compared to BACEBV-GFPWT. This supports a proliferative role for residues 130-159 of EBNA3C in the context of EBV-induced transformation of B cells.

Several independent studies from our group also showed that this region of EBNA3C specifically interacted with many important cellular proteins, such as the master regulator of apoptosis, p53 and its interacting proteins Mdm2, ING4 and ING5, as well as E2F1 and c-Myc [12, 17, 24]. We have previously shown that EBNA3C can also de-regulate p53 in stable cell lines as well as in EBV infected primary cells [18]. Further, we also showed that knock-down of EBNA3C during viral infection delayed the onset of p53 expression in human PBMCs [18]. These results provide a body of evidence showing that EBNA3C is able to directly regulate p53 down-regulation during EBV infection. Moreover the expression of p53 in response to BACEBV-GFPWT and delta EBNA3C virus infection of primary cells appears to have increased by the second day in the knockout virus compared to a decrease with the BACEBV-GFPWT. This result is similar to earlier studies from our group and others which showed higher expression of the p53 tumor suppressor on day 2. This led to a decrease in the production of virus-associated with primary B-cells infection during early stages of infection [17, 38].

The targeted degradation of p53 by one of its negative regulators, Mdm2, represents a critical circuit in the regulation of p53-mediated tumor suppressive functions [39]. In this line of evidence, we have recently shown that EBNA3C recruits the Mdm2-E3 ubiquitin ligase activity for enhancing proteasome dependent proteolysis of p53 [12]. Our current findings suggested that residues 130- 159 of EBNA3C is important for Mdm2 stabilization which serves to regulate p53. This study is undoubtedly a significant step towards our understanding of the importance of residues 130-159 of EBNA3C in development of EBV-associated human lymphomas and to design effective therapies targeting the p53-Mdm2 complex.

Tumor viruses have developed numerous sophisticated strategies to ensure continuous proliferation of latently infected cells [39]. The transition from one cell cycle phase to another is critically regulated by four major families of proteins, such as Cyclins, Cdks, Cdk inhibitors (CdkIs) and pocket proteins [40]. So far, nine Cdks and 16 Cyclins have been identified and a specific combination of a particular Cdk with its regulatory cyclin molecule forming an active complex is required for cell cycle progression at each stage [26, 41], and expression can lead to a significant upregulation of CyclinD1 without affecting its transcription [8]. EBNA3C plays a dual role in increasing nuclear localization of CyclinD1 by blocking its polyubiquitination, as well as inhibiting GSK-3b-mediated kinase activity [8]. CyclinD1 binding motif is located at the N-terminal region of EBNA3C (residues 130–159), a stretch of amino acid sequence that is also conserved in other −3C homologous proteins in both Baboon and Rhesus lymphocryptoviruses [9]. In the present study, we found that cells infected with EBVGFPΔE3C130-159 virus infected cells have lower expression of CyclinD1 compared to BACEBV-GFPWT infection. Therefore, these data suggests that this short stretch of EBNA3C domain is of particular significance in deregulating cell proliferation of EBV-infected cells through CyclinD1.

The role of the pRb-E2F pathway in the regulation of cell-cycle progression, particularly the G1-S transition, is well established [42]. Our previous report suggested that the N- and C-terminal domains of EBNA3C forms a stable pRb independent complex with the N-terminal DNA-binding region of E2F1 responsible for inducing apoptosis [17]. Both pRb and E2F1 may also play important roles in p53 and CyclinD1 mediated cell cycle regulation. Therefore, it is logical rationale to monitor the downstream effects of EBNA3C130-159 residues. Our study revealed that cells infected EBVGFPΔE3C130-159 differentially expressed E2F1 and pRb when compared to BACEBV-GFPWT infected cells suggesting the importance of these residues in regulating the pRb-E2F pathway.

p53 protects cells from malignant transformation by inducing either cell cycle arrest or apoptosis in response to persistent infection of multiple tumor viruses [43]. Earlier, we demonstrated that EBNA3C-expressing cells are resistant to apoptosis, which is consistent with the observation that EBNA3C promotes cell proliferation [8, 19]. Using both *in vitro* and *in vivo* binding experiments, we showed that p53 can physically interact with the N-terminal domain of EBNA3C [24]. This further emphasizes the importance of this critical domain of EBNA3C for bypassing cell cycle checkpoints in EBV-infected cells. Recently, a genetic study using conditionally active EBNA3C, showed that deletion of 130-159 particular domain was not able to maintain cell proliferation of EBV-transformed LCLs [23]. Our study now show that an EBV virus deleted for residues 130-159 of EBNA3C results in more apoptotic cell death when compared to wild type infected cells. We showed that PARP1 cleavage was enhanced along with an increase in the cell death population in the cells infected with this mutant virus.

**Figure 11:**
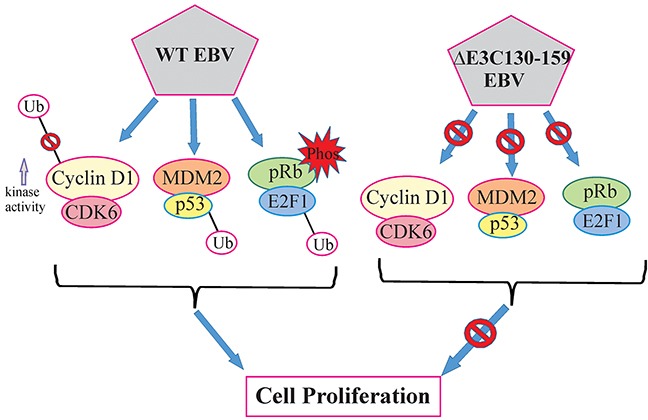
A model for EBNA3C residues 130-159 of EBV in B-cell proliferation The schematic shows the vital role of EBNA3C with CyclinD1/CDK6, MDM2/p53 and pRb/E2F1 binding site within EBNA3C for modulating cell signaling pathways which ultimately regulating cell proliferation.

In most cases, chromosomal instability results from numerous structural alterations such as micro and multi-nuclei formation [30]. Previous studies found that an inactivated p53 response may overcome cell cycle checkpoints and maintain proliferation regardless of the chromosomal aberrations [31, 32]. Our result showed that infection with EBV deleted for residues 130-159 can play a critical role in reducing chromosomal instability when compared to BACEBV-GFPWT infection. This data suggests that residues 130-159 within the N-terminus domain of EBNA3C is in large part responsible for inducing these abnormalities, which are closely related to its ability to counteract p53 transactivation. A schematic of the vital role of EBNA3C with CyclinD1/CDK6, MDM2/p53 and pRb/E2F1 binding site within EBNA3C for modulating cell signaling pathways which ultimately regulating cell proliferation has been shown in Figure 11.

In conclusion, we deleted residues 130-159 of EBNA3C using BACmid recombinant engineering technology. The growth assays demonstrated that deletion of the above residues within EBNA3C resulted in a reduction of these recombinants infected cells to drive cell proliferation. Interestingly, this recombinant exhibited a higher infectivity for human PBMCs. Further investigation revealed that deletion of 130-159 might also be able to differentially regulate the p53/Mdm2, CyclinD1/Cdk6 and pRb/E2F1 pathways when compared to BACEBV-GFPWT virus infection. Furthermore, deletion of the above domain had a dramatic effect on its ability to activate apoptosis in infected B-cells during early stages of infection. Therefore EBNA3C, and its association with p53 and CyclinD1 can contribute to growth and activation of B-cells. We also explored the regulation of the pRb/E2F1 signaling pathway in this recombinant virus infected cells. Our results suggest a contributory role of E2F1 and its interaction with EBNA3C to induction of pRb/E2F1 signaling important for B-cell transformation. This study provides new insights towards our understanding of the importance of residues 130-159 of EBNA3C in development of EBV-associated human lymphomas and to design effective therapies targeting its binding complexes.

## MATERIALS AND METHODS

### Cells and antibodies

Wild type and mutant viruses expressing HEK-293T cells were cultured in DMEM with 5% bovine growth serum (Gibco, Carlsbad, CA). De-identified PBMCs were provided from the Human Immunology Core at the University of Pennsylvania. The Core maintains an IRB approved protocol in which Declaration of Helsinki protocols were followed and each donor gave written informed consent. PBMCs were maintained in RPMI with 10% fetal bovine serum (FBS) (Hyclone, South Logan, Utah). A10 (EBNA3C), S12 (LMP1) hybridoma were described previously [23,44]. BZLF1 antibody was provided by Martin Rowe, (University of Birmingham, UK) [45]. EBNA1 antibody was purchased from Advanced Biotechnologies, Inc., Columbia, MD. Mouse monoclonal anti-p53 antibody (DO-1), mouse monoclonal antibody reactive to Mdm2 (SMP14) and Mouse antibodies to CyclinD1 (DSC-6) antibodies were purchased from Santa Cruz Biotechnology, Inc (Santa Cruz, CA), GAPDH antibody was obtained from US-Biological Corp. (Swampscott, MA).

### Constructs of BACEBV-GFP mutations

BACEBV-GFP were constructed as described previously [16]. Our protocol was based a method using positive and negative selection of the galactokinase expression cassette (*galk*) [20]. The *galk* cassette was amplified from plasmid p*galK* using primers 50 bp upstream and downstream of 130-159 aa domain of the EBNA3C ORF. Further, 100 bp double strand oligo used to remove the *galk* cassette. The primers and oligoes sequence used for constructing EBV mutation are listed in Supplementary Table S1. The deletion was confirmed by restriction enzyme analysis followed by Southern blot analysis. The clone were further confirmed by junction PCR followed by sequencing.

### Induction and purification of recombinant virus

Construct of BACEBV-GFP mutation were transfected into HEK-293T cells via CaPO_4_ method. Puromycin (1μg/ml) was then added for selection 24 hrs after transfection. Three weeks after selection, homogenous populations of GFP-positive cells harboring EBV episomes were obtained. The stable cell lines were confirmed by visualization of GFP protein by immunofluorescence. Butyric Acid at a final concentration of 3 mM and TPA (Sigma) at 20 ng/ml was used for lytic induction [16]. Cell suspensions were centrifuged at 3000 rpm for 20 min and the supernatant was filtered through a 0.45 μm cellulose acetate filter. The viral particles were concentrated by ultracentrifugation at 70,000xg at 4°C and stored at −80°C.

### Infection of PBMCs with recombinant EBV virions

Infection of PBMCs were performed as described previously [46]. In brief, 1×10^7^ were infected by incubation with virus suspension in 1ml of RPMI 1640 (with 10% FBS) medium in the presence of Polybrene at a final concentration 5 ng/μl (Sigma, Marborough, MA) and incubated for 4 hrs in 37°C. Cells were centrifuged for 5 min at 1500 rpm, the supernatant discarded, pelleted cells were washed by fresh RPMI medium for 2 times and resuspended in fresh RPMI 1640 (10% FBS) medium in 6-well plates and culture at 5% CO2, 37°C humidified incubator.

### Extraction and determination of intracellular and extracellular viral DNAs

For quantitation of intracellular viral DNA, cells were harvested and washed twice with 1xPBS to remove the residual viruses. Extracellular viral DNA was extracted from culture supernatants as described previously [46, 47]. In brief, virions were pellet down at 70,000xg for 2 hrs at 4°C and resuspended. Cellular DNAs and free viral DNAs were removed by treatment with DNase I at 37°C for 1~2 hrs. Virion DNA was treated with HMW buffer for 20 minutes. Lysates were treated with proteinase K overnight at 37°C with subsequent extraction with phenol/chloroform/isopropanol. Intracellular and extracellular viral DNAs were quantitated by real-time DNA PCR for EBNA1 (5′-CATTGAGTCGTCTCCCCTTTGGAAT-3′ and 5′-TCATAACAAGGTCCTTAATCGCATC-3′). DNA from the Namalwa cell line was used as a standard by which EBV genomic DNA was measured.

### Cell growth assay

BACEBV-GFPWT and EBVGFPΔE3C130-159 were transfected into HEK-293T cells. The cells were monitored and selected to stable cell lines with puromycin (1μg/ml). 1,000 cells per samples were seeded in 12 cm Petri dish in DMEM supplemented with 5% BGS. After 1, 2, 3,4 and 5 days, cells were fixed on the plates with 4% formaldehyde and stained with 0.1% crystal violet. The amount of the colonies in each dish was scanned by an Odyssey scanner (LI-COR Biosciences, Lincoln, NE) and the cell density was quantitated using the Odyssey V3.0 software.

### Cell proliferation assay

1×10^5^ BACEBV-GFPWT and EBVGFPΔE3C130-159 expressing HEK-293T cells were plated into each well of the 6-well plates and grown in DMEM medium for 6 days at 37°C incubation. Viable cells were counted daily using an automated cell counter by Trypan blue dye exclusion technique.

### RT-PCR

Total RNAs and cDNAs were prepared as described previously [48]. RT-qPCR was performed on a StepOnePlus real-time PCR System (Applied Biosystems Inc, Carlsbad, CA). The reactions were carried out in a 96-well plate at 95°C for 10 min, followed by 35 cycles at 95°C for 20 s, 52°C for 30 s and then 72°C for 30 s. The differences of cycle threshold values (CT) between the samples (ΔCT) were calculated after standardization by GAPDH and converted to fold changes using one of the samples as a standard (1-fold). The Primers used for qRT-PCR were listed in Supplementary Table S1.

### Immunoblotting

Cells were lysed with RIPA buffer (10mM Tris, pH 7.5, 1% Nonidet-P40, 2mM Na_2_EDTA, 150mM NaCl plus protease inhibitors) and protein estimation was performed by Bradford assay. The lysates were analyzed by Western blots using appropriate primary antibodies and infrared-tagged secondary antibodies. The results were scanned with an Odyssey Infrared scanner. Densitometry analysis was performed with the Odyssey V3.0 software.

### Immunofluorescence

Cells were collected and fixed to the slides with 4% paraformaldehyde with 0.1% Triton X-100 for 20 min, and subsequently blocked with 10% BSA at room temperature for 30 min. Cells were then incubated with a primary antibody (Mouse anti-EBNA1 or EBNA3C, Rabbit anti-BZLF1), and specific signals were detected with a secondary antibodies conjugated with Alexa Fluor 488, 594 or 647 (Invitrogen, Carlsbad, CA). The cells were counterstained with 4′, 6′-diamidino-2-phenylindole (DAPI). The results were analyzed with a Fluoview FV300 microscope (Olympus Inc., Melville, NY).

### Apoptosis assay

For apoptosis measurement, we used a mixture of ethidium bromide (EB) and acridine orange (AO) dye (1:1). The dye mixture contained 100 μg/ml AO and 100 μg/ml EtBr in 1×PBS. Infected BJAB cells after 5 days were treated with 12 hrs of serum starvation with etoposide. The medium was aspirated and washed with 1× PBS twice. Subsequently, the dye mix (EB-AO) was added and left for 5 min, followed by two PBS washes. The slides were monitored for green, red, and orange colors using the appropriate filters. Cells were observed and counted by fluorescence microscopy. All assays were done in triplicate, counting a minimum of 400 total cells each to plot a graph for the apoptotic index. The infected BJAB cells were subjected to further analysis for cleavage of PARP1 and Caspase 3 antibodies.

### Detection of chromosome instability

The number of cell nuclei was determined by staining cells with the DNA dye 4′, 6′-diamdino-2-phenylindole (DAPI). BJAB cells uninfected and infected with both BACEBV-GFPWT and EBVGFPΔE3C130-159 virus were washed with PBS and spread evenly on a slide. The cells were fixed with 1:1 methanol-acetone for 10 min at −20°C and then stained with DAPI at 1μg/ml for 10 min at room temperature. Slides were examined with an Olympus 1×70 fluorescence microscope. Multinucleation and micronucleation were examined with at least 2×10^2^ cells from three randomly selected fields and counted to determine the percentage and to obtain the standard deviation for each sample.

### Cell cycle assay

Infected PBMCs cells were harvested, washed three times with PBS, and fixed with a 1:1 ratio of methanol-acetone for 12 hrs at 4°C. Further, cells were incubated with 200 μg/ml of RNase A and kept at −20°C for 3 hrs. The cells were stained with propidium iodide (PI) (40 μg/ml; Sigma, St. Louis, MO) in PBS for at least 1 hrs at 4°C in the dark. Cells at different cell cycle phases with appropriate controls were differentiated using FACSCalibur (BD Biosciences, San Jose, CA), and the results were assessed with the FlowJo software (TreeStar, Ashland, OR).

### Statistical analysis

Data represented here as mean values with standard errors of the means (SEM). 2-tailed Student's t test was performed to evaluate the significance of differences in the mean values and P value <0.05 was considered as statistically significant.

## SUPPLEMENTARY FIGURE AND TABLE


