# Supplementary material for: An EBV recombinant deleted for residues 130-159 in EBNA3C can deregulate p53/Mdm2 and Cyclin D1/CDK6 which results in apoptosis and reduced cell proliferation
Source: Oncotarget. 2016 Feb 19;7(14):18116–34. doi: 10.18632/oncotarget.7502 (PMC4951276; doi:10.18632/oncotarget.7502)
Supplement: Supplementary file 1 [file oncotarget-07-18116-s001.pdf]

SUPPLEMENTARY FIGURE AND TABLE

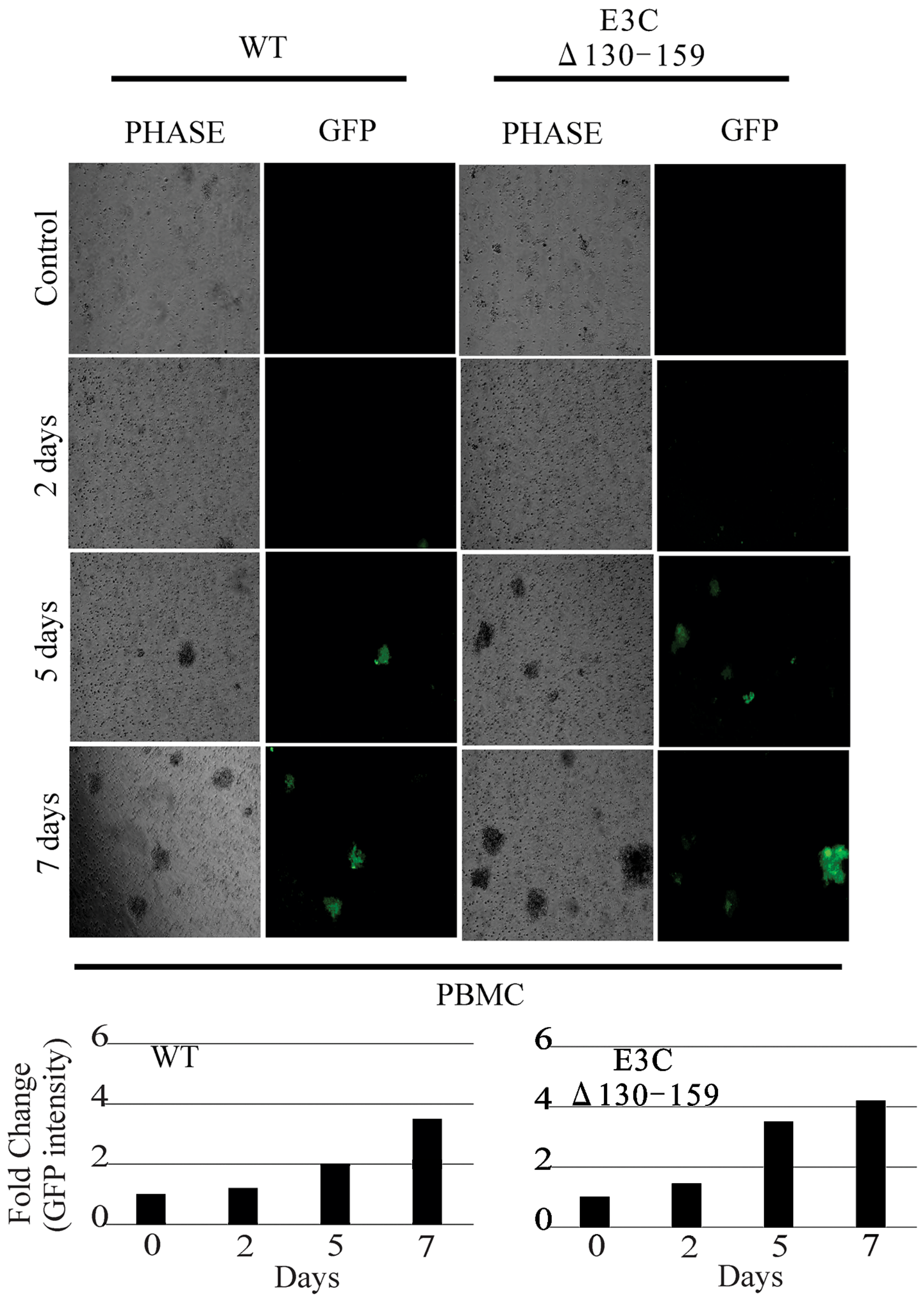

Supplementary Figure S1: BACEBV-GFPWT and EBVGFPΔE3C130-159 infection in PBMCs. PBMCs were infected with BACEBV-GFPWT (WT) and deleted residue of (EBVGFPΔE3C130-159) 130-159 for EBNA3C. Microscopy were performed in days dependent manner as there viruses have GFP signals. Quantitation of GFP plotted according to images represented in figure.

Supplementary Table S1: Primer and oligo sequences

**EBV delta E3C 130-159 galK insertion cassette [galk sequence Underlined]**	
Sense	ATGCTGCCTGCCGGGCTGTCAAGCAATCGCACCTGCAAGCG CTATCAAAC<u>CCTGTTGACAATTAATCATCGGCA</u>
Antisense	GGAGATGTTAGAAGCCAATGTCGCCAACCCACACCGCCCTC CGCAACCAA<u>TCAGCACTGTCCTGCTCCTT</u>
**EBV delta E3C 130-159 galK removal cassette [100 bp]**	
Sense	ATGCTGCCTGCCGGGCTGTCAAGCAATCGCACCTGCAAGCG CTATCAAACTTGGTTGCGGAGGGCGGTGTGGGTTGGCGACA TTGGCTTCTAACATCTCC
Antisense	GGAGATGTTAGAAGCCAATGTCGCCAACCCACACCGCCCTC CGCAACCAAGTTTGATAGCGCTTGCAGGTGCGATTGCTTGAC AGCCCGGCAGGCAGCAT
**EBNA3C 130-159 (Junction PCR)**	
Sense	GCATCAGGCGAAGGCGGAGAAGAC
Antisense	CTGGCTGCCATCACGAAGCACAAG
**EBNA-1**	
Sense	CATTGAGTCGTCTCCCCTTTGGAAT
Antisense	TCATAACAAGGTCCTTAATCGCATC
**P53**	
Sense	CCT GAGGTTGGCTCTGACTGTA
Antisense	TCCGTCCCAGTAGATTACCAC
**MDM-2**	
Sense	CCGGAATTCATGAGTGTGGAATCTAGTTTGC
Antisense	ATAAGAATGCGGCCGCGGGGAAATAAGTTAGC
**Cyclin D1**	
Sense	AGTTGTTGGGGCTCCTCAG
Antisense	TCTGGAGAGGAAGCGTGTGA
**pRb**	
Sense	CAGAAGGCAACTTGACAAGAGA
Antisense	CCTTCTCGGTCCTTTGATTG
**E2F1**	
Sense	GGCCAGGTACTGATGGTCA
Antisense	GACCCTGACCTGCTGCTCT
**GAPDH**	
Sense	TGCACCACCAACTGCTTAG
Antisense	GATGCAGGGATGATGTTC
